# The Functions of Crucial Cysteine Residues in the Arsenite Methylation Catalyzed by Recombinant *Human* Arsenic (III) Methyltransferase

**DOI:** 10.1371/journal.pone.0110924

**Published:** 2014-10-28

**Authors:** Shuping Wang, Zhirong Geng, Nan Shi, Xiangli Li, Zhilin Wang

**Affiliations:** State Key Laboratory of Coordination Chemistry, School of Chemistry and Chemical Engineering, Nanjing University, Nanjing, Jiangsu, PR China; St. Georges University of London, United Kingdom

## Abstract

Arsenic (III) methyltransferase (AS3MT) is a cysteine (Cys)-rich enzyme that catalyzes the biomethylation of arsenic. To investigate how these crucial Cys residues promote catalysis, we used matrix-assisted laser desorption ionization-time of flight-mass spectrometry (MALDI-TOF-MS) to analyze Cys residues in recombinant human arsenic (III) methyltransferase (hAS3MT). We detected two disulfide bonds, Cys250-Cys32 and Cys368-Cys369, in hAS3MT. The Cys250-Cys32 disulfide bond was reduced by glutathione (GSH) or other disulfide bond reductants before the enzymatic methylation of arsenite (iAs^3+^). In addition to exposing residues around the active sites, cleavage of the Cys250-Cys32 pair modulated the conformation of hAS3MT. This adjustment may stabilize the binding of *S*-Adenosyl-L-methionine (AdoMet) and favor iAs^3+^ binding to hAS3MT. Additionally, we observed the intermediate of Cys250-*S*-adenosylhomocysteine (AdoHcy), suggesting that Cys250 is involved in the transmethylation. In recovery experiments, we confirmed that trivalent arsenicals were substrates for hAS3MT, methylation of arsenic occurred on the enzyme, and an intramolecular disulfide bond might be formed after iAs^3+^ was methylated to dimethylarsinous acid (DMA^3+^). In this work, we clarified both the functional roles of GSH and the crucial Cys residues in iAs^3+^ methylation catalyzed by hAS3MT.

## Introduction

Arsenic has complex effects on the human body and is capable of functioning as both a cancer inducer and cancer therapy [1]–[5]. Evidence suggests that the biotransformation of arsenic is related to its biological functions [6]. The methylation of arsenite (iAs^3+^) catalyzed by arsenic (III) methyltransferase (AS3MT) has been generally used to investigate the biotransformation of arsenic in mammals [7]–[11]. Oxidative methylation and glutathione conjugation have been proposed as two possible mechanisms for the metabolic pathways of arsenic [12]–[14]. Recent *in* *vitro* observations have strongly suggested that arsenic is methylated in trivalent forms [15]. Our previous work evaluating iAs^3+^ methylation catalyzed by recombinant human arsenic (III) methyltransferase (hAS3MT) suggested that the valence state of arsenic was not changed in the transmethylation [16], [17]. The enzymatic methylation of arsenic is a reductant-related reaction. The reductants that can initiate the hAS3MT-catalyzed iAs^3+^ methylation are those disulfide bond reductants such as tris (2-carboxyethyl) phosphine hydrochloride (TCEP), dithiothreitol (DTT), Glutathione (GSH), cysteine (Cys), and β-mercaptoethanol [16], [17]. Mechanistic studies of the interaction between reactants and hAS3MT have suggested that *S*-Adenosyl-L-methionine (AdoMet), reductant and iAs^3+^ sequentially bound to hAS3MT before the enzymatic methylation. In this pathway, the reductant increased the number of exposed Cys residues and perturbed the conformation of hAS3MT [16].

Protein Cys residues are structurally and functionally important [18]–[21]. Some Cys residues participate in disulfide bonds to stabilize the native conformation of protein and maintain protein integrity [20]. Some Cys residues sense oxidizing factors and easily form disulfide bonds in response. These Cys residues and their corresponding disulfide bonds usually serve as a redox-sensing switch to modulate protein function [19]–[21]. Cys residues are also frequently used at active sites to bind metals [22]. Active site Cys residues of enzymes always promote the thiol-based redox catalysis by forming intramolecular or extramolecular disulfide bonds [23].

AS3MT is a Cys-rich enzyme. Relative to other amino acids, Cys residues are critical for the enzymatic methylation of arsenic [12], [13]. Fomenko *et al.* suggested that the redox-active Cys157 and Cys207 are the active site residues of mouse AS3MT. These residues promoted thiol-based catalysis by forming an intramolecular disulfide bond at Cys157-Cys207 [23]. Rosen *et al.* investigated the structure of CmArsM, an ortholog of hAS3MT that was isolated from the thermoacidophilic eukaryotic red alga *Cyanidioschyzon merolae*. This group identified Cys224, Cys174, and Cys72 as the binding sites of iAs^3+^. A disulfide bond may be formed between Cys72 and Cys174. However, Cys72 was not involved in the second methylation step to convert monomethylarsonous acid (MMA^3+^) to dimethylarsinous acid (DMA^3+^) [24], [25]. The conserved Cys residues proposed to participate in catalysis of mouse AS3MT and CmArsM correspond to Cys206, Cys156 and Cys61 in hAS3MT [23], [25]. There are fourteen Cys residues in hAS3MT. Thomas *et al.* and our group have analyzed the functions of these crucial Cys residues and have confirmed that Cys61, Cys156, and Cys206 are the active sites of hAS3MT [26]–[30]. Like Cys72 in CmArsM, Cys61 moves toward Cys156 and Cys206 upon AdoMet binding, and leaves away after the first step methylation [30]. However, it is not clear if a disulfide bond is formed between the active residues of during the catalytic cycle. Cys250 is critical to the structure of hAS3MT, the enzyme loses its catalytic activity when Cys250 is replaced with serine [29]. The roles of critical Cys residues in the catalytic activity of hAS3MT thus remain in need of further study.

In this work, we studied the structure and function of hAS3MT to investigate the iAs^3+^ methylation. Two disulfide bonds, Cys250-Cys32 and Cys368-Cys369, were detected in hAS3MT by matrix-assisted laser desorption ionization-time of flight-mass spectrometry (MALDI-TOF-MS). GSH, including other disulfide bond reductant, reduced the Cys250-Cys32 pair before the catalytic cycles. Fluorescence spectra suggested that Cys250-Cys32 cleavage may favor the binding of AdoMet and iAs^3+^. We also detected the intermediate of Cys250-*S*-adenosylhomocysteine (AdoHcy), this suggests that Cys250 is involved in the methylation. Moreover, the results of high performance liquid chromatography-inductively coupled plasma (HPLC-ICP)-MS showed that the methylation of trivalent arsenicals occurred on hAS3MT and that reductant recovered the catalytic activity of hAS3MT.

## Materials and Methods

Caution: Handling arsenic compounds requires safeguards to mitigate any potential risk [1], [6].

### Preparation of hAS3MT and mutants

The modified gene for hAS3MT was cloned into the *Bam*HI-*Sal*I restriction sites of pET-32a vector (Novagen, USA). The plasmid pET-32a-*hAS3MT* was verified by DNA sequencing, and the hAS3MT protein was expressed from *E. coli* BL21(DE3) pLysS at 25°C [27]. Site-directed mutagenesis was directly performed on the cDNA encoding for hAS3MT in the pET-32a-*hAS3MT* plasmid. The primers used for mutagenesis were described previously [28], [29]. The obtained mutants of C250S and C360S, in which Cys was respectively replaced with serine, were confirmed by DNA sequencing. Mutants were expressed from *E. coli* BL2(DE3) pLysS at 20°C. The obtained enzymes were separated on a 2.5 mL nickel nitrilotriacetic acid agarose column (Novagen, USA) by imidazole buffer. Target proteins were dialyzed four times against phosphate buffer (PBS) (25 mM, pH 7.0) with 0.05 mM DTT to remove imidazole. Dialysis buffer was prepared before use and completely degassed. Details were published previously [27]–[29].

### Sample preparation for MALDI-TOF-MS

Concentration of the purified protein was determined by Bradford assay using a bovine serum albumin standard [31]. Target proteins were concentrated to 1.0 µg/µl and then loaded onto a 12% non-reducing sodium dodecyl sulfate-polyacryl amide gel for electrophoretic separation. The band corresponding to hAS3MT or its mutants was excised and washed twice with 50 mM ammonium acetate buffer (pH 6.0). The bands containing the target proteins were then treated with 20 mM IA buffer (pH 6.0) in the dark at room temperature for 1.5 h. After alkylation, samples were washed twice with 50 mM ammonium acetate buffer (pH 6.0) and incubated in 5% trypsin buffer (pH 6.0) overnight at 37°C. Subsequently, peptide fragments in digested gel pieces were obtained by sonicating three times for 5 min in 60% acetonitrile and 0.1% (v/v) trifluoroacetic [16], [32]. Samples were lyophilized and stored at −80°C for mass spectrometry analysis. All buffers used in sample preparation were completely degassed.

Before mass analysis, lyophilized samples were dissolved in H_2_O. 1.0 µL of the supernatant was loaded on an AnchorChip target and dried in air. Then 0.8 µL of a-cyano-4-hydroxycinnamic acid matrix solution (0.5 µg/mL with freshly prepared solution containing 90% ACN and 0.1% TFA) was applied to the same spot and left to dry for 2 min. Mass spectrometry was performed on an AUTOFLEX II MALDI-TOF mass spectrometer (Bruker, Germany) with a 20-kV acceleration voltage.

### Cys residues analysis

C360S, hAS3MT, and their reduced forms were prepared for determination of Cys residues. Reduced hAS3MT and C360S were prepared by incubating the proteins with 100 mM DTT at 37°C for 60 min before gel separation [19]. The functions of crucial Cys residues were analyzed by detecting Cys residues after hAS3MT-catalyzed the iAs^3+^ methylation in TCEP, GSH, DTT, and Cys reaction systems. Reaction systems containing hAS3MT (4.0 µM), a reductant (TCEP (0.7 mM), GSH (7.0 mM), DTT (1.5 mM), or Cys (10 mM), AdoMet (1.0 mM), and iAs^3+^ (1.0 µM), were incubated at 37°C for 60 min, then immediately lyophilized for gel separation. The effects of TCEP, GSH, DTT, Cys, and AdoMet on hAS3MT were studied by incubating hAS3MT with 0.7 mM TCEP, 7.0 mM GSH, 1.5 mM DTT, 10.0 mM Cys, or 1.0 mM AdoMet at 37°C for 60 min before gel separation. The separated proteins were alkylated, digested, and analyzed by MADLDI-TOF-MS [15].

### Fluorescence spectroscopy

Experiments were performed on a 48,000 DSCF time-resolved fluorescence spectrometer (SLM Co., USA). At room temperature, three-dimensional fluorescence spectra were evaluated to study the conformation of hAS3MT and C250S [16]. The excitation wavelength (Ex) of protein (2.0 µM) was recorded form 200 nm to 340 nm, and the emission wavelength (Em) was recorded from 230 nm to 500 nm. Fluorescence quenching spectra of C250S and hAS3MT were used to study the binding models of AdoMet. The experiments were performed by titrating AdoMet into enzyme preparations (2.0 µM) at 27°C or 37°C. The corresponding data were recorded, Em was measured from 300 to 450 nm and Ex fixed at 276 nm.

### Recovery experiments of TCEP and DTT

Arsenicals were analyzed by HPLC-ICP-MS (PRP X-100, Hamilton/Elan9000). The conformations of hAS3MT were determined on a JASCO-J810 Spectropolarimeter spectroscopy (Jasco Co., Japan). The secondary structure parameters of hAS3MT were computed by Jwsse32 software with reference of CD-Yang. Jwr [33].

After incubated at 37°C for 60 min, the GSH reaction system, containing hAS3MT (2.0 µM), GSH (7.0 mM), AdoMet (1.0 mM), and iAs^3+^ (2.0 µM), was dialyzed against phosphate buffer as a control for further experiments. The recovery effects of TCEP and DTT on the iAs^3+^ methylation were determined by analyzing the arsenic species on hAS3MT. In the experiments, 10 µL PBS, DTT (0.05 M), or TCEP (0.07 M) were respectively added to 1.0 mL dialyzed control containing 0.1 mM AdoMet, then incubated at 37°C for another 60 min. Arsenicals in the recovery systems were oxidized by H_2_O_2_ and analyzed by HPLC-ICP-MS [17], [34]. Meanwhile, adding of TCEP and DTT induced conformational changes that were studied by CD at room temperature.

## Results

### Two disulfide bonds are detected in hAS3MT

The structural characteristics of hAS3MT are closely related to its function in iAs^3+^ methylation [25], [28], [29]. However, the reduced state or disulfide state of the crucial Cys residues in hAS3MT is still unclear. To address this question, we studied these Cys residues in hAS3MT by MALDI-TOF-MS.

**Figure 1:**
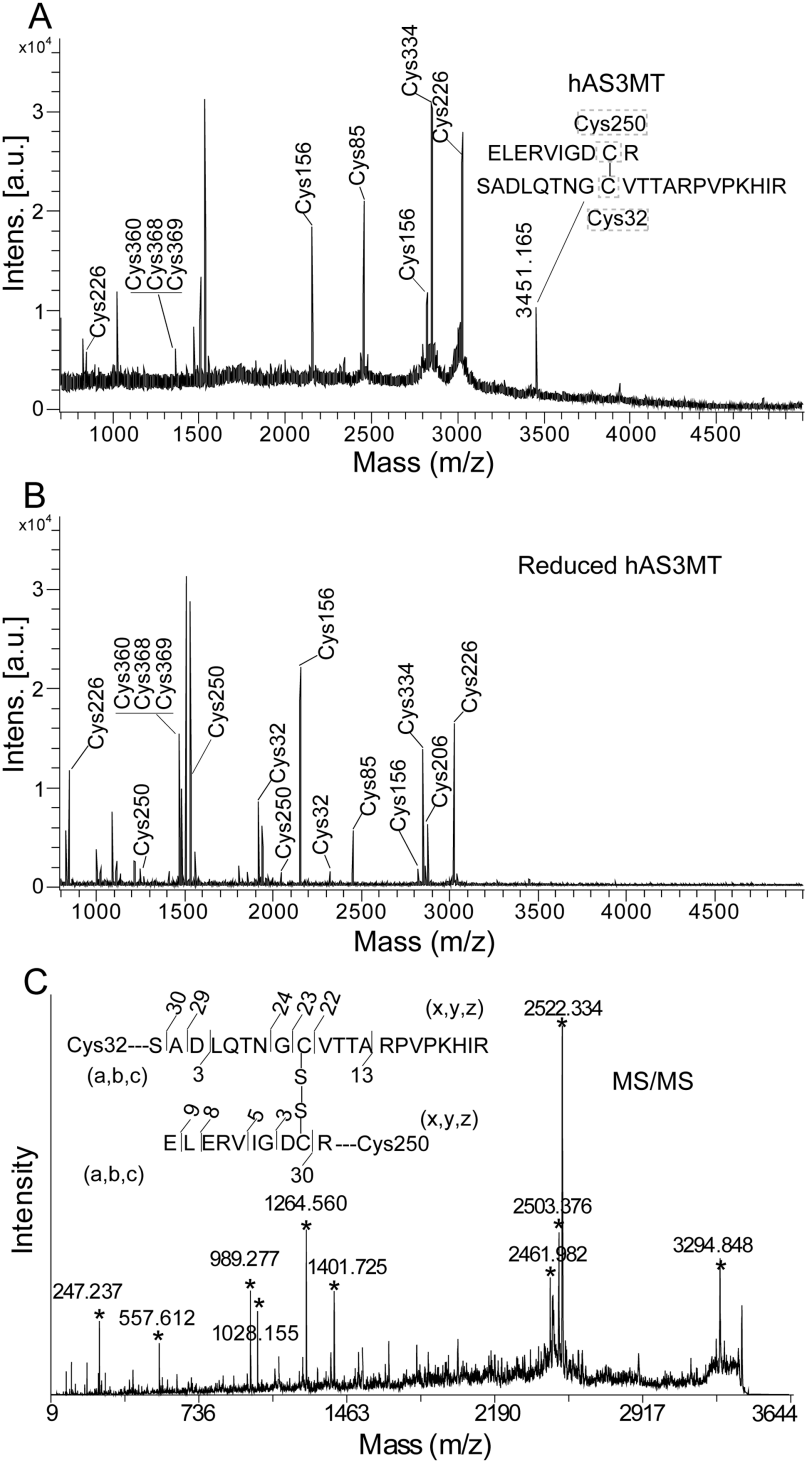
MALDI-TOF-MS spectra of the tryptic digests of hAS3MT (A) and its reduced form (B). (C) MS/MS spectra for the sequence at 3451.17 (m/z). Labels a, b, c, x, y, and z represent the cleavage manners of peptides.

Seven peptides containing iodoacetamide (IA)-modified Cys residues, corresponding to Cys85, Cys156, Cys226, Cys334, Cys360, Cys368 and Cys369, were detected in hAS3MT by MALDI-TOF-MS (Figure 1a and Table 1). When hAS3MT was reduced with 100 mM DTT, we observed seven more peptides containing IA-modified Cys residues at 250, 206 and 32 (Figure 1b and Table 2). Moreover, the peak at 3451.17 (m/z) disappeared when hAS3MT was reduced by DTT (Figure 1b). After further analysis, we deduced that this peak corresponded to a peptide containing the Cys250-Cys32 disulfide bond. To confirm this observation, we used MALDI-TOF MS/MS to analyze the fragments of the peptide corresponding to 3451.17 (m/z). This analysis confirmed the existence of the Cys250-Cys32 pair (Figure 1c and Table 3) [35].

**Table 1:** Sequences for the peptide fragments of hAS3MT analyzed by MALDI-TOF-MS.

Sequence	site	Observed	Mr (expt)	Mr (calc)	ppm
DCYVLSQLVGEKGHVTGIDMTK	84–105	2450.28	2449.27	2449.20	28.58
LGEAGIKNESHDIVVSNCVINLVPDK	139–164	2820.89	2819.88	2819.45	152.49
NESHDIVVSNCVINLVPDK	146–164	2152.30	2151.29	2151.06	106.91
ELAVLAQKIGFCPPRLVTANLITIQNK	215–241	3008.05	3007.04	3006.71	109.74
IGFCPPR	223–229	846.31	845.30	845.42	−141.96
FAQDFLIRPIGEKLPTSGGCSALELK	315–340	2847.73	2846.72	2846.50	77.28
CVPDAAGGCCGTKK	360–373	1366.62	1365.61	1365.58	21.97

Values of observed M+H^+^ (Da) are the results of MALDI-TOF-MS in Figure 1a.

**Table 2:** Sequences for the peptide fragments of reduced hAS3MT analyzed by MALDI-TOF-MS.

Sequence	site	observed	Mr (expt)	Mr (calc)	ppm
SADLQTNGCVTTARPVPKHIR	24–44	2320.91	2319.90	2320.21	−133.63
SADLQTNGCVTTARPVPK	24–41	1914.72	1913.71	1913.96	−130.64
DCYVLSQLVGEKGHVTGIDMTK	84–105	2449.86	2448.85	2449.20	−142.92
LGEAGIKNESHDIVVSNCVINLVPDK	139–164	2819.93	2818.92	2819.45	−188.02
NESHDIVVSNCVINLVPDK	146–164	2151.82	2150.81	2151.06	−116.24
THKVLWGECLGGALYWKELAVLAQK	198–222	2870.89	2869.89	2869.53	125.44
ELAVLAQKIGFCPPRLVTANLITIQNK	215–241	3007.99	3006.98	3006.71	89.79
IGFCPPR	223–229	846.27	845.26	845.42	−189.29
ELERVIGDCRFVSATFR	242–258	2054.82	2053.81	2054.04	−111.99
ELERVIGDCR	242–251	1246.42	1245.41	1245.61	−160.59
VIGDCRFVSATFR	246–258	1527.54	1526.53	1526.77	−157.22
FAQDFLIRPIGEKLPTSGGCSALELK	315–340	2847.06	2846.05	2846.50	−158.11
CVPDAAGGCCGTKK	360–373	1480.74	1479.74	1479.63	74.34

Values of observed M+H^+^ (Da) are the results of MALDI-TOF-MS in Figure 1b.

**Table 3:** Sequences for Cys32-Cys250 peptide fragments at 3451.17 (m/z) analyzed by MALDI-TOF-MS/MS.

Sequence	calculated	observed	ppm
SAD	247.25	247.24	−40.45
IGDCR	557.63	557.61	−35.87
ELERVIGDC	989.16	989.28	121.30
LERVIGDCR	1028.23	1028.16	−68.08
SADLOTNGCVTTA	1264.41	1264.56	118.62
VTTARPVPKHIR	1401.69	1401.73	28.54
ERVIGDCR–GCVTTARPVPKHIR	2461.96	2461.98	8.12
LERVIGDCR–CVTTARPVPKHIR	2503.06	2503.38	127.83
DCR–ADLQTNGCVTTARPVPKHIR	2521.98	2522.33	138.76
ELERVIGDCR–DLQTNGCVTTARPVPKHIR	3294.82	3294.85	9.11

Values of observed M+H^+^ (Da) are the results of MALDI-TOF-MS/MS in Figure 1c. Mass for each fragments were calculated according to the standard fragmentation pattern.

Previous studies have suggested that Cys72 may be important for the maintenance of hAS3MT conformation [29], and Cys61 may be the third binding site for iAs^3+^ [30]. However, we did not detect the peptides corresponding to Cys61 (IA-modified) and Cys72 (IA-modified) in hAS3MT or its reduced form. Combined with the hAS3MT structural model, based on the crystal structure of CmArsM, we hypothesized that Cys61 and Cys72 might be buried intemally, the in gel IA-modification and trypsin digestion may not be complete [25], [30].

**Figure 2:**
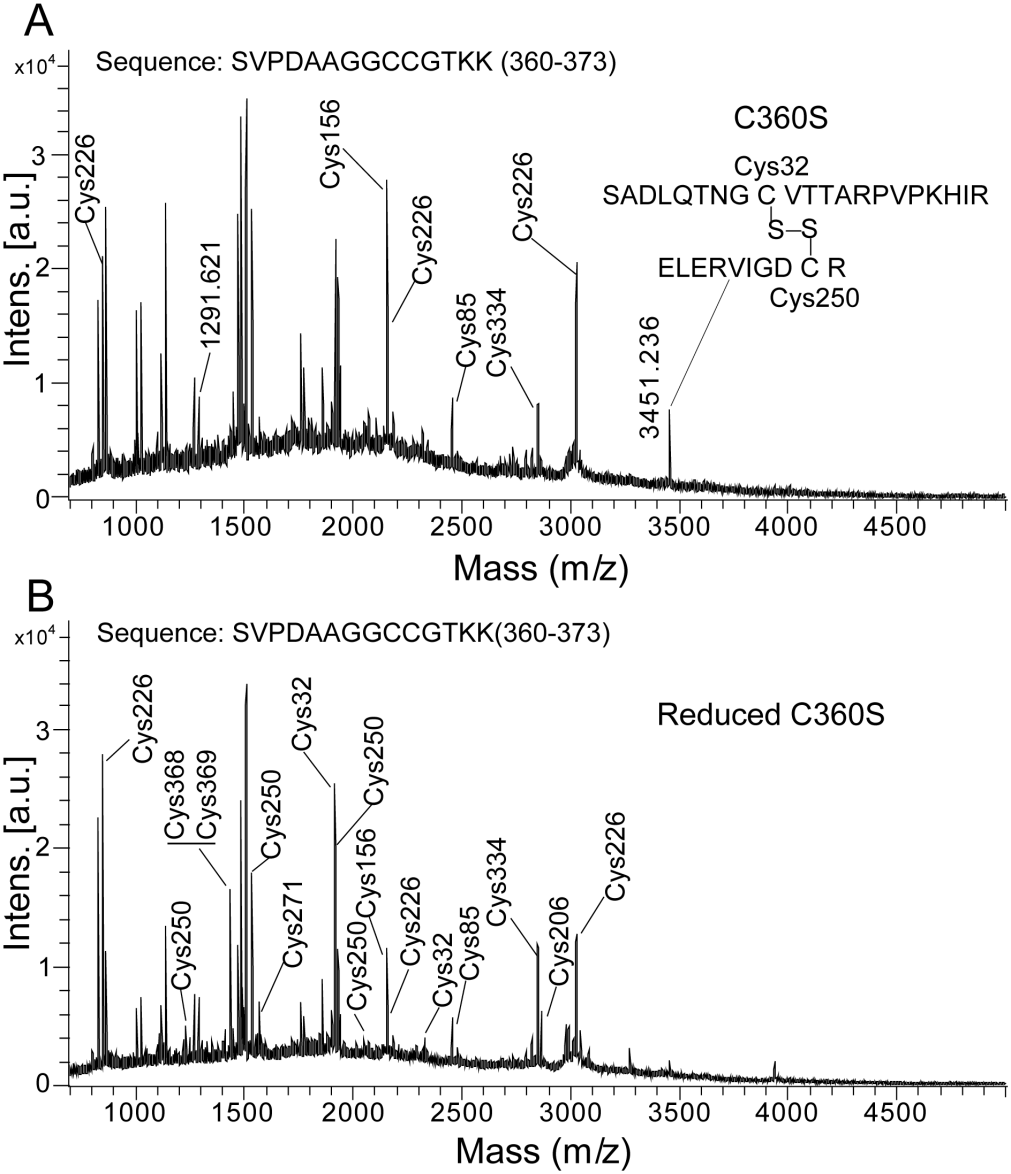
MALDI-TOF-MS spectra of the tryptic digests of C360S (A) and its reduced form (B).

When hAS3MT was reduced with 100 mM DTT, we also observed that the relative intensity of the peak corresponding to IA-modified Cys360, Cys368 and Cys369 was markedly increased, and the mass of the peak shifted from 1366.62 (m/z) to 1480.74 (m/z) (Figure 1, Table 1 and Table 2). Previous studies have suggested that Cys368 and Cys369 may exist as a Cys-Cys pair [26], [29]. Therefore, we hypothesized that this pair was cleaved by 100 mM DTT. To obtain the direct evidence for the existence of the Cys368-Cys369 pair, we used site-directed mutagenesis to replace the Cys at 360 with serine, and analyzed the Cys residues at C360S. As shown in Figure 2, the mutation at Cys360 did not induce the cleavage of Cys32-Cys250 disulfide bond. The peak corresponding to Cys368 and Cys369 (IA-modified) in C360S was not detected, but the peak corresponding to SVPDAAGGCCGTKK at 1291.62 (m/z) was obviously detected (Figure 2a). Meanwhile, the peak corresponding to Cys368 and Cys369 (IA-modified) at 1406.89 (m/z) was detected when C360S was reduced by DTT (Figure 2b). These results proved the existence of the Cys368-Cys369 disulfide bond in hAS3MT.

### Cys250-Cys32 pair is reduced before iAs^3+^ methylation

Enzymatic methylation of iAs^3+^ is a reductant-regulated reaction. Recent studies have suggested that GSH, DTT, TCEP, and Cys can promote the hAS3MT-catalyzed iAs^3+^ methylation *in* *vitro* [16], [17]. Therefore, the four disulfide bond reductants were selected to investigate the function of key Cys residues in iAs^3+^ methylation.

**Figure 3:**
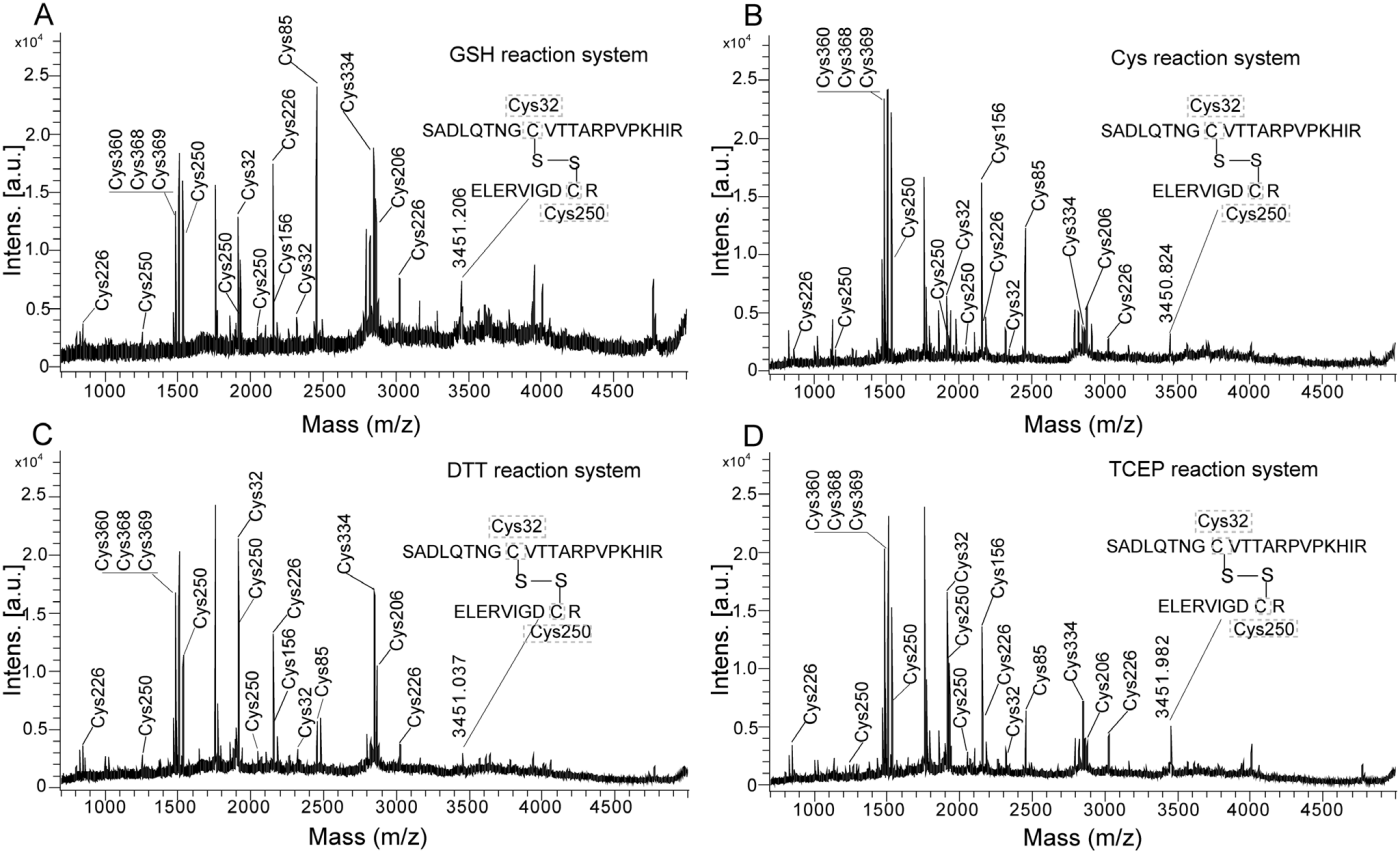
MALDI-TOF-MS spectra for the digested hAS3MT after it catalyzed the iAs^3+^ methylation in GSH (A), Cys (B), DTT (C), or TCEP (D) reaction system. Before analysis, hAS3MT catalyzed the iAs^3+^ methyation in GSH, Cys, DTT, or TCEP reaction system at 37°C for 60 min.

After hAS3MT catalyzed the iAs^3+^ methylation in GSH (7 mM) reaction system at 37°C for 60 min, Cys residues were analyzed by MALDI-TOF-MS. The intensity of the peak corresponding to Cys250-Cys32 at 3451.21 (m/z) was decreased, and the peaks corresponding to Cys250 (IA-modified), Cys32 (IA-modified) and Cys206 (IA-modified) were detected (Figure 3a and Table 4). Similar changes in Cys residues were observed after hAS3MT-catalyzed the iAs^3+^ methylation in DTT (1.5 mM) (Figure 3b), TCEP (0.7 mM) (Figure 3c), or Cys (10 mM) (Figure 3d) reaction system. The Cys250-Cys32 disulfide bond of hAS3MT was cleaved in the iAs^3+^ methylation.

**Table 4:** Sequences for the IA-modified peptide fragments of Cys32 and Cys250 after hAS3MT-catalyzed the iAs^3+^ methylation.

Sequence site	Mr (calc)	GSH system		Cys system		DTT system		TCEP system	
		Mr (expt)	ppm	Mr (expt)	ppm	Mr (expt)	ppm	Mr (expt)	ppm
24–44	2320.21	2320.15	−25.86	2319.71	−215.54	2320.08	−56.03	2319.96	−107.76
24–41	1913.96	1914.18	114.93	1914.23	141.05	1914.13	88.81	1913.87	−47.03
242–258	2054.04	2054.35	150.90	2054.34	146.03	2054.31	131.43	2053.81	−111.99
242–251	1245.61	1245.78	136.46	1245.52	−72.26	1245.72	88.30	1245.39	−176.65
246–261	1915.01	1914.89	−62.67	1915.12	57.44	1915.99	−15.67	1915.04	15.67
246–258	1526.77	1526.69	−52.40	1526.65	−78.60	1526.71	−39.3	1526.58	−124.46

Values of Mr (expt) are calculated from the results of MALDI-TOF-MS in Figure 3. ND represents the peptide fragment is not detected.

**Figure 4:**
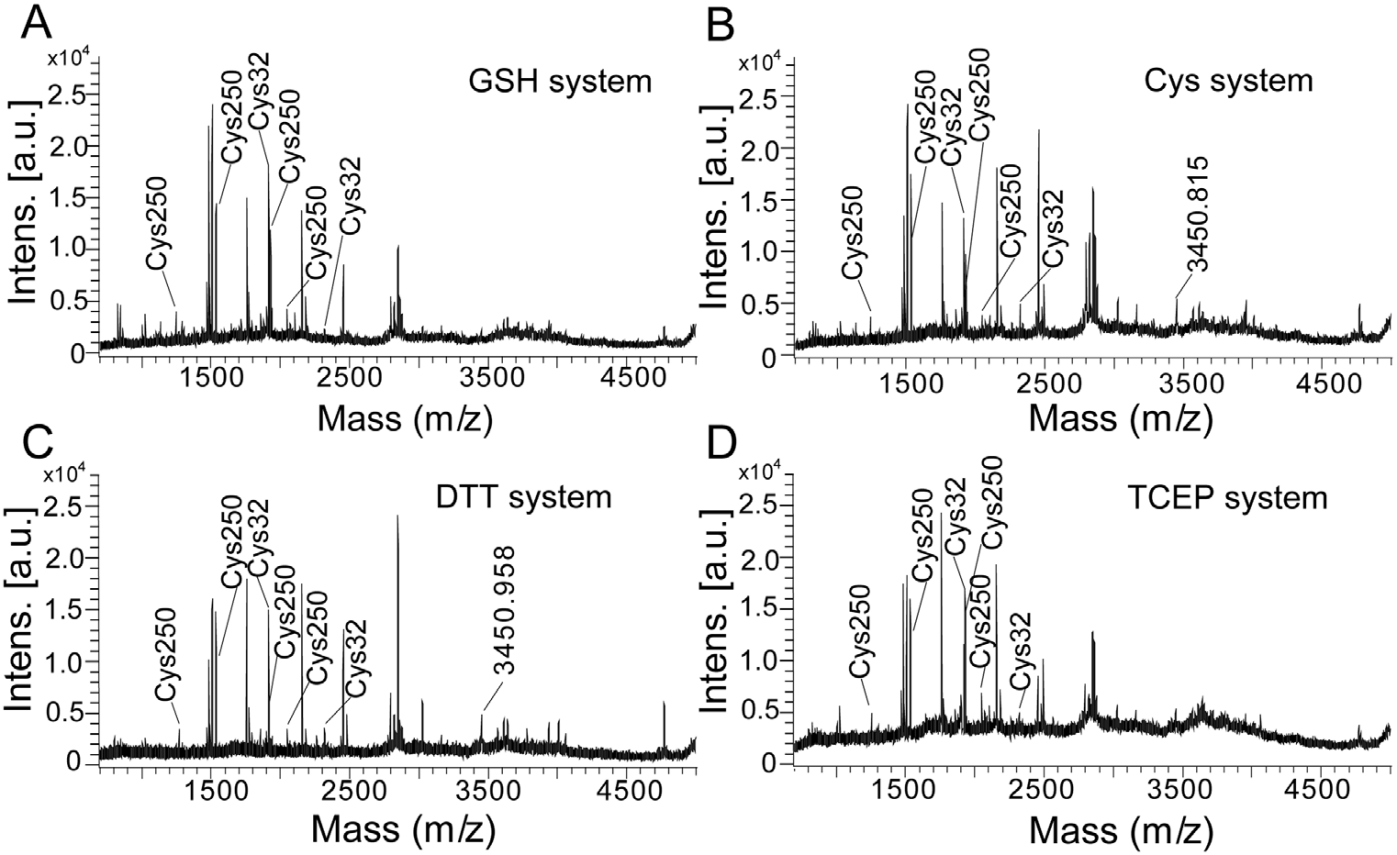
The effects of (A) GSH, (B) Cys, (C) DTT, and (D) TCEP on the Cys250-Cys32 disulfide bond. Before analysis, hAS3MT was respectively reduced by GSH (7 mM), Cys (10 mM), DTT (1.5 mM) or TCEP (0.7 mM) for 60 min at 37°C.

After reduced by GSH (7.0 mM), DTT (1.5 mM), TCEP (0.7 mM) or Cys (10 mM), hAS3MT was analyzed by MALDI-TOF-MS to determine whether the Cys250-Cys32 disulfide bond was reduced before the catalytic cycles. As shown in Figure 4a, when hAS3MT was incubated with GSH for 60 min at 37°C, the peak corresponding to the Cys250-Cys32 pair at 3451.21 (m/z) disappeared. Meanwhile, peaks corresponding to Cys250 (IA-modified) and Cys32 (IA-modified) were detected. These changes in Cys residues proved that the disulfide bond of Cys250-Cys32 was reduced by GSH before the iAs^3+^ methylation. Peaks corresponding to Cys250 (IA-modified) and Cys32 (IA-modified) were also detected, and the intensity of the peak corresponding to the Cys250-Cys32 disulfide bond was decreased when hAS3MT was reduced by Cys (Figure 4b), DTT (Figure 4c), or TCEP (Figure 4d). In summary, the Cys250-Cys32 pair of hAS3MT was reduced by reductant before the catalytic cycles.

### Cys250 is involved in the methyl releasing step

**Figure 5:**
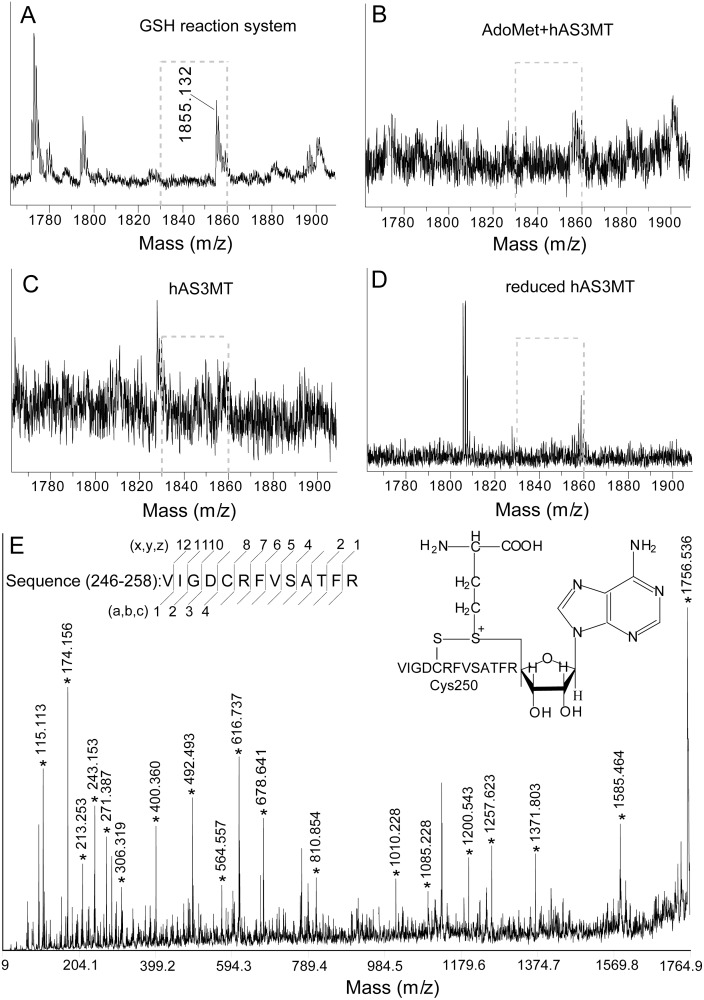
The intermediate of Cys250-AdoHcy analyzed by MALDI-TOF-MS. (A) in GSH reaction system. (B) in AdoMet control. (C) in hAS3MT. (D) in reduced hAS3MT. (E) MS/MS spectra for the sequence at 1855.13 (m/z) of hAS3MT in GSH reaction system. Labels a, b, c, x, y, and z represent the cleavage manners of peptide. AdoMet control: 1.0 mM AdoMet was incubated with 2.0 µM hAS3MT at 37°C for 60 min.

Our group has previously studied the role of Cys residues and shown that Cys250 is critical to hAS3MT-catalyzed methylation of iAs^3+^ [29]. After hAS3MT catalyzed the iAs^3+^ methylation in GSH (7.0 mM) reaction system for 60 min, we detected a new peak at 1855.13 (m/z) by MALDI-TOF-MS (Figure 5a). This peak was also detected after hAS3MT-catalyzed the iAs^3+^ methylation in Cys, TCEP and DTT reaction systems (data not shown). However, we did not find this peak after incubation hAS3MT with 1.0 mM AdoMet at 37°C for 60 min (Figure 5b). Additionally, the peak at 1855.13 (m/z) was not detected in hAS3MT (Figure 5c) or its reduced form (Figure 5d). We hypothesized that this peak at 1855.13 (m/z) may be an intermediate of AdoHcy and the sequence corresponding to VIGDCRFVSATFR at 246–258. Because the disulfide bond of AdoHcy-Cys250 would be reduced by reductant and cleaved by other reasons in the midst of mass analysis, the signal to noise of the peak at 1855.13 (m/z) was low. To confirm the presence of this intermediate, we analyzed the peak at 1855.13 (m/z) by MALDI-TOF-MS/MS (Figure 5e and Table 5). Fragment peaks corresponding to AdoHcy-Cys250 were discerned. These results suggested that the peak at 1855.13 (m/z) was an intermediate of Cys250-AdoHcy. Cys250 participated in iAs^3+^ methylation.

**Table 5:** Sequences for the AdoHcy-Cys250 peptide fragments at 1855.13 (m/z) analyzed by MALDI-TOF-MS/MS.

Sequence	calculated	observed	ppm	Sequence	calculated	observed	ppm
V	115.13	115.11	−173.75	VSATFR	678.76	678.64	−176.82
R	174.19	175.16	−172.25	FVSATFR	810.94	810.85	−111.00
VI	213.29	213.25	−187.57	RFVSATFR	1010.13	1010.23	98.99
VIG	271.34	271.39	184.24	CRFVSATFR	1085.27	1085.23	−36.86
FR	306.37	306.32	−163.23	DCRFVSATFR	1200.36	1200.54	149.93
VIGD	400.43	400.36	−174.84	GDCRFVSATFR	1257.41	1257.62	166.98
ATFR	492.55	492.49	−121.83	IGDCRFVSATFR	1371.57	1371.80	167.66
SATFR	564.63	564.56	−123.99	DCRFVSATFR-AdoHcy	1585.36	1585.46	63.07
VIGDCR	616.77	616.74	−48.64	IGDCRFVSATFR-AodHcy	1756.57	1755.54	−17.08

Values of observed M+H^+^ (Da) are the results of MALDI-TOF-MS/MS in Figure 5e. Mass for each fragments were calculated according to the standard fragmentation pattern.

### Mutation of Cys250 exposes the active sites of hAS3MT

Beyond exposing Cys250, the cleavage of Cys250-Cys32 may change the conformation of hAS3MT. To simulate the conformational changes of hAS3MT induced by cleavage of the Cys250-Cys32 pair, we used site-directed mutagenesis to substitute the Cys residue at 250 with serine.

**Figure 6:**
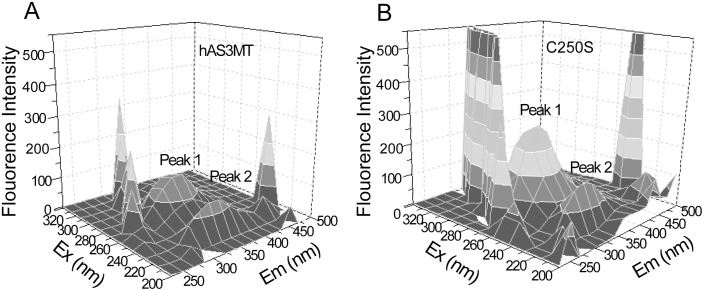
Three-dimensional fluorescence spectra of (A) hAS3MT and (B) C250S. Each experiment was carried out three times.

Three-dimensional fluorescence was used to study the changes around tryptophan (Trp) residues. The three Trp residues (Trp73, Trp203, and Trp213) of hAS3MT are near the crucial Cys residues (Cys32, Cys61, Cys72, Cys156, Cys206, and Cys250) [16], [27], [30]. Two peaks are shown in the three-dimensional fluorescence spectrum of hAS3MT (Figure 6a). Peak 1 represents the microenvironments around Trp and tyrosine residues near the active sites of hAS3MT. Peak 2 refers to the fluorescence of polypeptide backbone structures [36], [37]. Compared to hAS3MT, the peak 1 fluorescence intensity of C250S was dramatically increased and blue-shifted, while peak 2 of C250S did not obviously change (Figure 6b). Mutation of Cys250 resulted in more residues near Trp being exposed on the protein surface, but did not appreciably perturb the polypeptide backbone of hAS3MT [16], [36].

### Cys250 mutation favors AdoMet binding to hAS3MT

**Figure 7:**
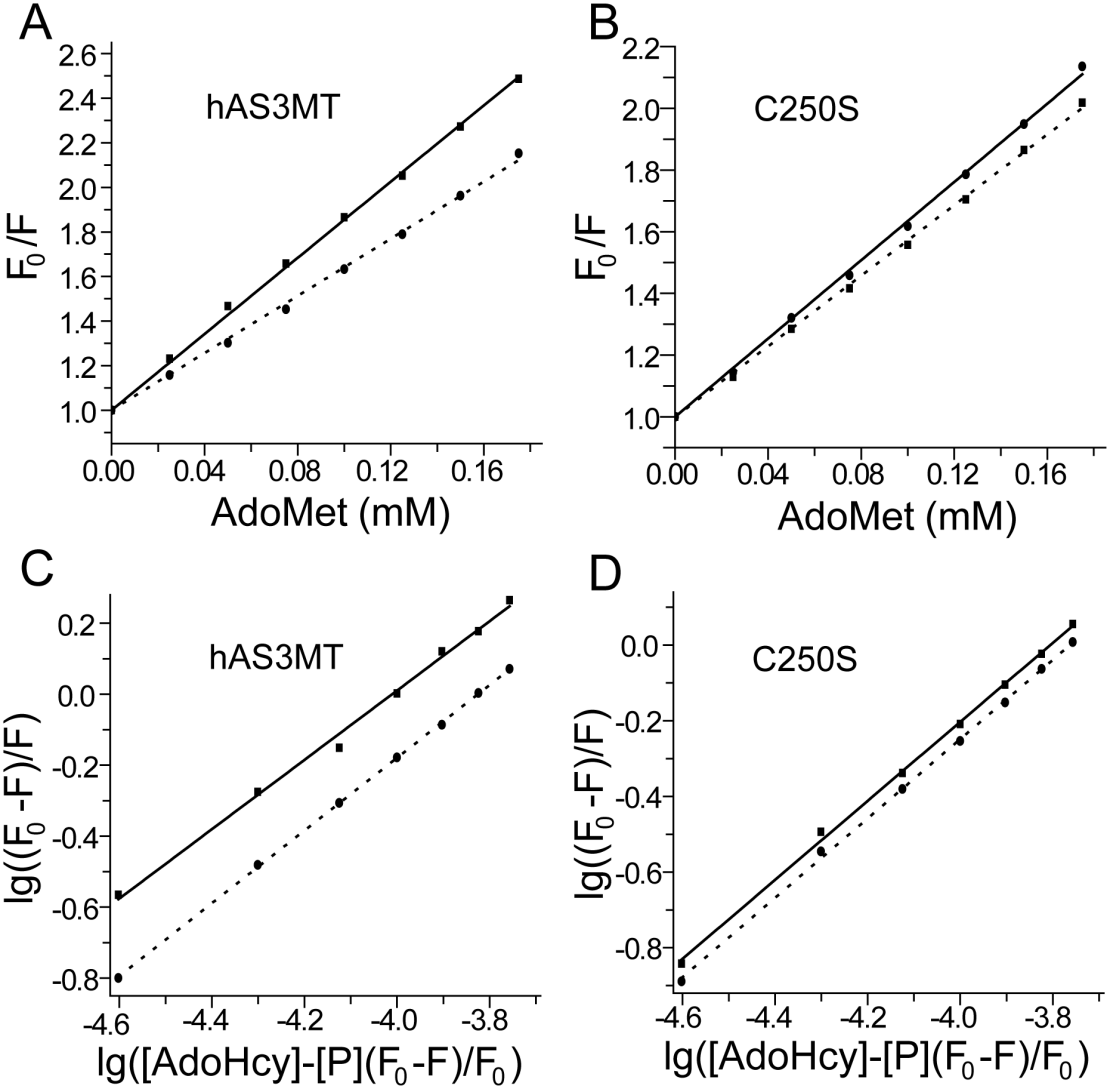
Fluorescence quenching of hAS3MT/C250S with AdoMet. (A, B) Stern-Volmer curves and (C, D) Double logarithm curves for the fluorescence quenching of hAS3MT and C250S with AdoMet at 350 nm, Ex: 276 nm. Solid line and dot line respectively represent the temperature at 27°C and 37°C. Each experiment was carried out three times.

To simulate whether Cys250-Cys32 cleavage influences the binding of AdoMet, we used fluorescence quenching spectra to study the interactions between AdoMet and C250S/hAS3MT [16]. The Stern-Volmer curves showed that AdoMet induced static quenching of the fluorescence intensity in hAS3MT and C250S (Figures 7a and b) [16], [37]. Corresponding parameters such ΔH, ΔG and ΔS were calculated according to the equation proposed by Bi et al. (Figures 7c and d) [38]. The three parameters, ΔG<0, ΔS>0, and ΔH = 0.53 kJ/mol, suggested that AdoMet bound to hAS3MT by hydrophobic and electrostatic interactions (Table 6) [16]. However, the interaction between AdoMet and C250S was electrostatic where the value of ΔH is −6.59 kJ/mol. Mutation of Cys250 increases the electrostatic interactions for AdoMet binding, which should favor the positive charge AdoMet binds to its domain [25], [34], [39]. Therefore, the cleavage of Cys250-Cys32 might make AdoMet bind to hAS3MT more firmly.

**Table 6:** Thermodynamic parameters for the binding of AdoMet to hAS3MT/C250S.

Parameter	ΔG (kJ/mol)		ΔS(J/mol•k)	ΔH (kJ/mol)
	27°C	37°C		
hAS3MT	−22.55±3.57	−23.09±2.79	76.76±7.02	0.53±0.05
C250S	−22.86±2.69	−23.24±3.01	54.12±5.38	−6.59±1.33

Parameters are calculated by the data in Figure 7c and d using origin 8.0.

### Reductant recovers the catalytic activity of hAS3MT

To clarify whether disulfide bonds are formed between the active sites in the catalytic cycle, we designed recovery experiments. HPLC-ICP-MS was used to detect the trivalent arsenic species on hAS3MT after they were oxidized to pentavalent forms by H_2_O_2_. CD was used to analyze the secondary structure of hAS3MT.

**Figure 8:**
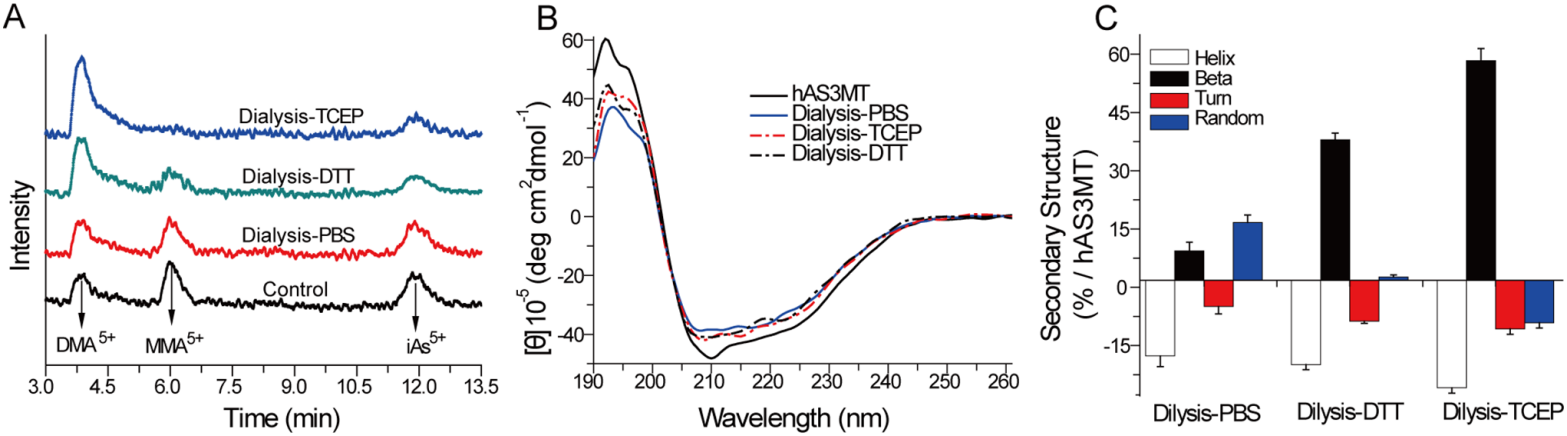
Recovery effects of TCEP and DTT on the enzymatic methylation of iAs^3+^. (A) HPLC-ICP-MS spectra of arsenic species on hAS3MT. Control: hAS3MT (2.0 µM) catalyzed the iAs^3+^ methylation in the GSH reaction system for 60 min and dialysis against PBS. Dialysis-PBS, Dialysis-DTT (0.5 mM), and Dialysis-TCEP (0.7 mM): PBS, DTT, or TCEP separately incubated with the dialyzed hAS3MT in the presence of AdoMet (0.1 mM) at 37°C for another 60 min. (B) CD spectra and (C) corresponding secondary structure of hAS3MT. Enzyme: hAS3MT (2.0 µM) in PBS. *Error bars* represent S.D. from the mean of three independent experiments.

As shown in control, after hAS3MT-catalyzed the As^3+^ methylation in GSH reaction system for 60 min, trivalent arsenic species bound to hAS3MT were not dialyzed out of the reaction (Figure 8a). When the dialyzed control was incubated with AdoMet (0.1 mM) at 37°C for another 60 min, the peaks corresponding to monomethylarsonic acid (MMA^5+^), dimethylarsinic acid (DMA^5+^) and inorganic pentavalent arsenic (iAs^5+^) were unchanged. However, incubation of the dialyzed hAS3MT with DTT (0.5 mM) or TCEP (0.7 mM) in the presence of AdoMet (0.1 mM) resulted in a recovery of iAs^3+^ methylation. Especially in the TCEP recovery system, the yield of DMA^5+^increased dramatically (Figure 8a). We found that the catalytic activity of hAS3MT was lost after dialysis. Use of reductant could recover the activity of hAS3MT and promote the on enzyme transfer of methyl groups from AdoMet to iAs^3+^ and MMA^3+^ [16], [40], [41]. Meanwhile, DTT and TCEP perturbed the conformation of dialyzed hAS3MT (Figure 8b). The secondary structure of hAS3MT, determined by CD, showed that DTT and TCEP increased the content of β-pleated sheets and decreased the number of α-helices (Figure 8c).

Hitherto, many studies of AS3MT-catalyzed iAs^3+^ methylation have proposed that intramolecular disulfide bonds may be formed between the active-site Cys residues in catalysis [17], [23], [25]. Therefore, the activity of crucial Cys residues was lost after hAS3MT catalyzed the iAs^3+^ methylation. When GSH was dialyzed out of the reaction system, hAS3MT failed to further catalyze the on-enzyme methylation. Because TCEP or DTT can reduce the formed disulfide bonds to recover the activity of crucial Cys residues, the on enzyme methylation occurred again. However, the active site Cys residues involved in the formation of disulfide bond were unclear.

## Discussion

AS3MT is a Cys-rich enzyme that contains four domains, including an arsenic binding domain and an AdoMet binding domain [25], [27], [29]. Because of the pro-sulfydryl properties of trivalent arsenicals, Cys residues play key roles in iAs^3+^ methylation [42]. Research on hAS3MT has suggested that Cys61, Cys156 and Cys206 are the three active sites of iAs^3+^ [27], [30]. Cys250 is crucial for the conformation and catalytic activity of hAS3MT [17], [27]. However, how these crucial Cys residues promote the enzymatic methylation of iAs^3+^ is unclear. Herein, we used MALDI-TOF-MS to investigate the functions of these Cys residues in the catalysis.

In our experiments, two disulfide bonds, Cys250-Cys32 and Cys368-Cys369, were detected in hAS3MT. We found that the Cys250-Cys32 disulfide bond was cleaved by GSH or other reductants before the catalytic cycle. The cleavage and formation of disulfide bonds always perturbs the conformation of proteins [18]–[21]. Therefore, the cleavage of Cys250-Cys32 induced conformational changes in hAS3MT was simulated by C250S. The results suggested that the cleavage of Cys250-Cys32 pair may expose residues around the active sites and make AdoMet bind to hAS3MT more firmly. As the second ordered reactant [16], reductant modulated the conformation of hAS3MT by reducing the disulfide bond of Cys250-Cys32, stabilizing the binding of AdoMet, and exposing active sites for iAs^3+^ binding. Thus, reductant transfers hAS3MT from an inactive state to an active state. Unlike Cys61, Cys156 and Cys206, the results of MALDI-TOF-MS suggested that Cys250 is involved in the transmethylation. Cys250 likely attacks AdoMet to transfer methyl to iAs^3+^ and bind with the generated AdoHcy in the form of Cys250-AdoHcy.

Studies on mouse AS3MT have suggested that the surface-exposed Cys157 and Cys207 serve as iAs^3+^ binding sites to promote the thiol-based redox catalysis. An intramolecular disulfide bond between Cys157 and Cys207 is likely formed during the catalytic cycles [23]. Previous studies on hAS3MT have suggested that a disulfide bond may be formed between Cys156 and Cys206 at the end of the catalytic cycle [17]. Studies on the structure of CmArsM regarded that Cys72 and Cys174, corresponding to Cys61 and Cys156 of hAS3MT, may form a disulfide bond when AdoMet is bound [25]. Although we have not acquired the direct evidence, recovery experiments suggest that a disulfide bond may be formed between the crucial Cys residues. We hypothesize that Cys156 and Cys206 may form an intramolecular disulfide bond in the catalysis. On the one hand, it is hardly denied the fact that the Cys72-Cys174 disulfide bond of CmArsM may be formed during the crystallization [25]. Moreover, the intramolecular disulfide bond of hAS3MT should be formed after iAs^3+^ is methylated to DMA^3+^ [17]. Cys61 leaves away from Cys156 and Cys206 in this pathway [25], [30]. On the other hand, Cys156 and Cys206 are surface-exposed on the β-pleated sheet of hAS3MT [16], [17], [27]. The cleavage of disulfide bond between Cys156 and Cys206 may increase β-pleated sheet content.

**Figure 9:**
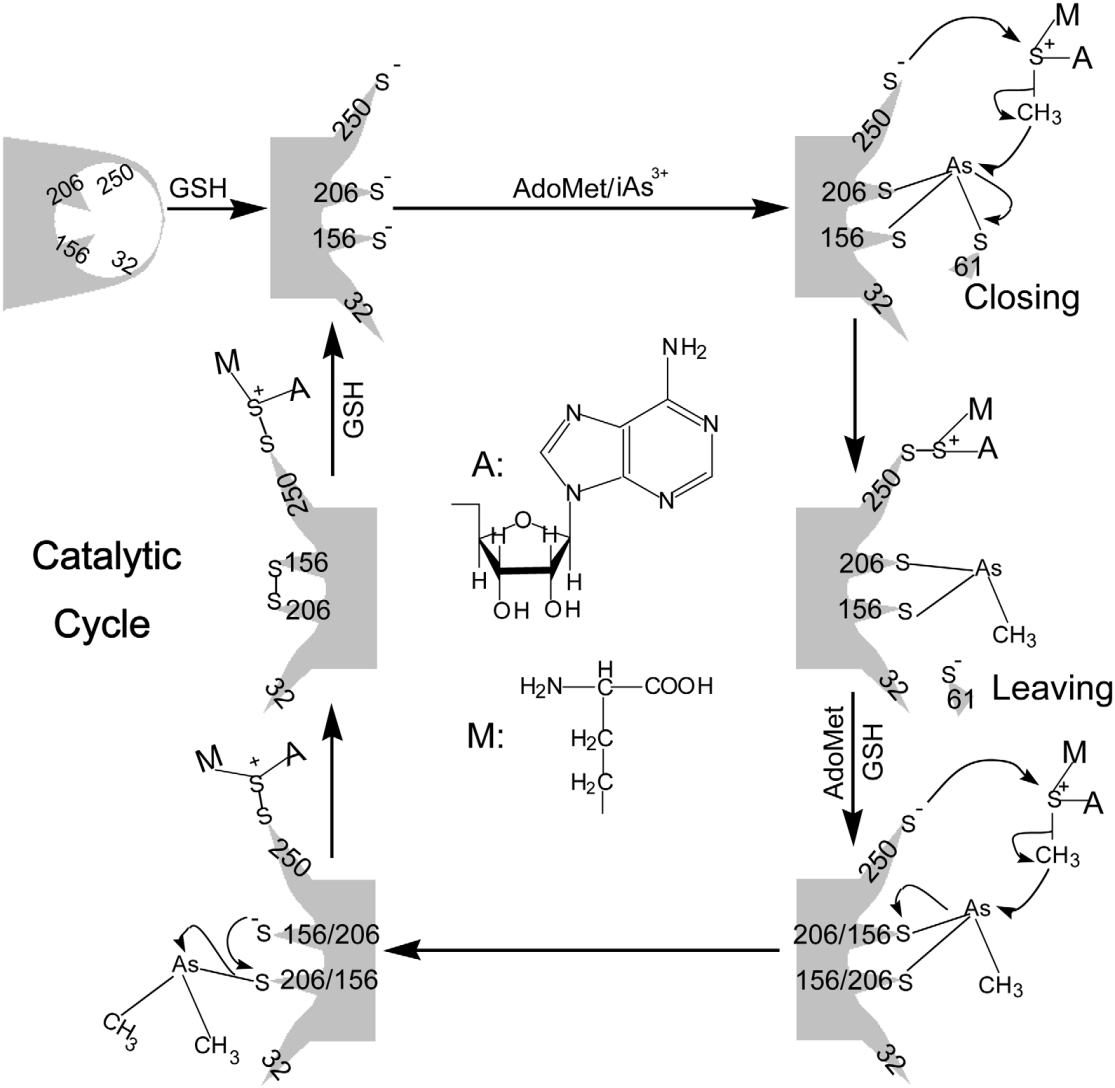
Possible model for the mechanism of hAS3MT-catalyzed iAs^3+^ methylation. A and M respectively represent the structure of Adenosyl and CH_2_CH_2_CH(NH_2_)COOH.

Although the purified hAS3MT may have different properties from the *in* *vivo* situation, studying the functions of crucial Cys residues is helpful to clarify the biotransformation of iAs^3+^. Herein, we proposed a model to clarify the hAS3MT-catalyzed iAs^3+^ methylation at molecular level. As shown in Figure 9, trivalent arsenicals are the substrates for hAS3MT. The on-enzyme arsenic methylation is occurred in the presence of GSH. After AdoMet binds to hAS3MT [16], GSH reduces the Cys32-Cys250 disulfide bond to expose Cys206, Cys250 and other residues. In this process, the conformation of hAS3MT is modulated by AdoMet and GSH. Meanwhile, Cys61 moves toward Cys156 and Cys206, and iAs^3+^ binds to the active sites for the on-enzyme methylation [24], [25], [30]. In the catalytic cycle, Cys250 is involved in the methyl transfer from AdoMet to iAs^3+^. After iAs^3+^ is methylated to MMA^3+^, Cys61 leaves away Cys156 and Cys206. GSH recovers the catalytic activity of Cys250 by reducing the Cys250-AdoHcy disulfide bond. Subsequently, the on-enzyme MMA^3+^ is methylated to DMA^3+^. Cys156 and Cys206 release DMA^3+^ away from the enzyme and form an intramolecular disulfide bond. GSH reduces the disulfide bonds formed at Cys250-AdoHcy and Cys156-Cys206 to recover the activities of these Cys residues for next cycle.

## Conclusions

In this work, we analyzed Cys residues in hAS3MT to investigate the mechanism for iAs^3+^ methylation. We found that the enzymatic methylation of arsenic occurred on the enzyme. GSH reduced the Cys250-Cys32 disulfide bond to release the Cys250 involved in the transmethylation. Moreover, we proposed a possible mechanism for the enzymatic methylation of iAs^3+^ and clarified the functions of GSH and crucial Cys resides in the catalytic cycles.
